# Cellular and acellular ex vivo lung perfusion preserve functional lung ultrastructure in a large animal model: a stereological study

**DOI:** 10.1186/s12931-018-0942-5

**Published:** 2018-12-04

**Authors:** Jasmin Steinmeyer, Simon Becker, Murat Avsar, Jawad Salman, Klaus Höffler, Axel Haverich, Gregor Warnecke, Christian Mühlfeld, Matthias Ochs, Anke Schnapper-Isl

**Affiliations:** 1 Institute of Functional and Applied Anatomy, Hannover Medical School, Hannover, Germany; 2 REBIRTH Cluster of Excellence, Hannover, Germany; 3 Department of Anesthesiology, Intensive Care, Palliative Care and Pain Medicine, BG University Hospital Bergmannsheil, Ruhr University Bochum, Bochum, Germany; 4 Department of Cardiothoracic, Transplantation and Vascular Surgery (HTTG), Hannover Medical School, Hannover, Germany; 5 Biomedical Research in Endstage and Obstructive Lung Disease Hannover (BREATH), Member of the German Center for Lung Research (DZL), Hannover, Germany

**Keywords:** Ex vivo lung perfusion, Ischaemia-reperfusion injury, Ischaemia ex vivo reperfusion associated lung injury, Lung ultrastructure, Stereology

## Abstract

**Background:**

Ex vivo lung perfusion (EVLP) is used by an increasing number of transplant centres. It is still controversial whether an acellular or cellular (erythrocyte enriched) perfusate is preferable. The aim of this paper was to evaluate whether acellular (aEVLP) or cellular EVLP (cEVLP) preserves functional lung ultrastructure better and to generate a hypothesis regarding possible underlying mechanisms.

**Methods:**

Lungs of 20 pigs were assigned to 4 groups: control, ischaemia (24 h), aEVLP and cEVLP (both EVLP groups: 24 h ischaemia + 12 h EVLP). After experimental procedures, whole lungs were perfusion fixed, samples for light and electron microscopic stereology were taken, and ventilation, diffusion and perfusion related parameters were estimated.

**Results:**

Lung structure was well preserved in all groups. Lungs had less atelectasis and higher air content after EVLP. No significant group differences were found in alveolar septum composition or blood-air barrier thickness. Small amounts of intraalveolar oedema were detected in both EVLP groups but significantly more in aEVLP than in cEVLP.

**Conclusions:**

Both EVLP protocols supported lungs well for up to 12 h and could largely prevent ischaemia ex vivo reperfusion associated lung injury. In both EVLP groups, oedema volume remained below the level of functional relevance. The group difference in oedema formation was possibly due to inferior septal perfusion in aEVLP.

**Electronic supplementary material:**

The online version of this article (10.1186/s12931-018-0942-5) contains supplementary material, which is available to authorized users.

## Background

Donor organ shortage is a main factor that limits the number of lung transplantations in patients with end-stage lung disease [1]. Every approach to enlarge the potential pool of donor lungs must ensure safe transplantation without increasing the risk of primary graft dysfunction (PGD) or other severe complications after transplantation. Ischaemia-reperfusion (IR) injury of the graft is a main reason for development of PGD [2]. In vivo IR injury and related ischaemia ex vivo reperfusion associated lung injury (IER injury) manifest as structural lung damage including oedema formation and injury of the blood-air barrier (bab) and results in impaired lung function [3–6].

Ex vivo lung perfusion (EVLP) offers the possibility to evaluate marginal lungs after procurement but before transplantation and to include suitable organs in the donor pool. Furthermore, lungs can be reconditioned during EVLP and first attempts for therapeutic interventions during EVLP have been made [7–12].

Various different EVLP protocols are applied clinically [10, 13]. One of the controversies centres on using acellular (aEVLP) or cellular perfusate in EVLP (cEVLP), i.e. erythrocyte addition to perfusate. Previous studies mainly focusing on functional parameters gave differential and sometimes conflicting results regarding the superiority of any one of the techniques [13–17]. However, uncertainties about preferable strategies limit further expansion of EVLP usage.

We assessed specifically the factor erythrocyte addition and compared aEVLP and cEVLP in an otherwise identical setting using a large animal ex vivo ischaemia and ex vivo reperfusion model. To promote susceptibility to lung injury, lungs were challenged with prolonged ischaemia (24 h) followed by 12 h EVLP. A previous study using the same animals indicated similar outcome in aEVLP and cEVLP regarding oxygenation and ventilation parameters, but higher pulmonary artery pressure (PAP) and higher pulmonary vascular resistance in cEVLP which was explained by a higher viscosity of the cellular perfusate [15].

The aim of this paper was to evaluate whether aEVLP or cEVLP preserves functional lung ultrastructure better and to generate a hypothesis regarding possible underlying mechanisms. Therefore, we performed a quantitative structural analysis by design-based stereology with particular reference to parameters related to lung function and IER injury.

## Methods

### Animals

The study was conducted in accordance with the German animal protection law and with the European Communities Council Directive 2010/63/EU for the protection of animals used for experimental purposes. All experiments were approved by the responsible authority (Lower Saxony State Office for Consumer Protection).

Twenty female pigs (56.6 kg +/− 8.7 kg bodyweight) were assigned by balanced randomisation to 4 groups (*n* = 5 per group): control, ischaemia, aEVLP and cEVLP. Lungs of ischaemia, aEVLP and cEVLP groups were subjected to 24 h of cold ischaemia (4 °C), lungs of EVLP groups additionally to 12 h of normothermic EVLP.

### Lung explantation and setup of EVLP

Details of surgical procedures and EVLP setup were described in Becker et al. [15]. Briefly, the animals were anaesthetized and lungs were procured via median sternotomy. Intraoperatively, all animals were ventilated in a pressure-controlled mode (inspiratory pressure 1.3 kPa, positive end-expiratory pressure (PEEP) 0.5 kPa after thoracotomy). For assessment of in vivo static compliance, ventilation was switched to a volume-controlled mode (tidal volume 7 ml/kg bw, PEEP 0.5 kPa). Intraoperative ventilation parameters were controlled permanently. In all groups, an intraoperative recruitment manoeuvre was performed (inspiratory hold at 2.5 kPa for 3 consecutive breaths) before flush perfusion. Lungs were flush perfused (60 ml/kg Perfadex®; XVIVO Perfusion, Gothenburg, Sweden) and clamped at an airway pressure of 1.5 kPa for organ recovery. Explanted lungs were immersed in cold Perfadex®. In the cEVLP group, 1.5 l of blood was collected from the caudal vena cava (= inferior vena cava) of each pig. Blood was leucocyte depleted (Leucocyte filter, Pall medical, Portsmouth, UK), washed with saline solution (Cell Saver, Fresenius, Schweinfurt, Germany) and centrifuged (Sigma 4 K15 Laboratory Centrifuge, Sartorius AG, Göttingen, Germany; 2300 revolutions/min at 4 °C) to prepare erythrocyte concentrate.

After ischaemic challenge, lungs of aEVLP and cEVLP groups were transferred into the EVLP circuit. The protocol was based on Lund [10] and Toronto techniques [13] but adapted to yield a common protocol for both groups that differed only in the factor “erythrocyte addition” [15]. Lungs in the aEVLP group were perfused with normothermic STEEN Solution® (XVIVO Perfusion) plus supplements (500 mg methylprednisolone, 1000 mg meropenem, 10,000 IU heparin, 12 ml trometamol buffer per 1.5 l STEEN Solution®). In the cEVLP group, autologous, leucocyte-depleted erythrocyte concentrate was added to perfusate contents otherwise identical to the aEVLP group to yield a haematocrit of 12–13% [15]. Initiation of EVLP during the first hour included gradual increase of perfusion, organ warming to normothermia and ventilation start [13]. At steady state, the following perfusion and ventilation parameters were applied: perfusion flow 70 ml/kg bw/min, left atrium (LA) pressure 0.2–0.4 kPa, ventilation frequency 10/min, tidal volume (V_t_) 7 ml/kg bw, PEEP 0.5 kPa, FiO_2_ = 0.21 (FiO_2_ = 1 for oxygenation assessment). The settings for perfusion flow followed the Lund strategy [10] and yielded physiological pulmonary artery pressures. We used a closed EVLP circuit comparable to the Toronto technique and LA pressures were kept in the desired range by adjusting the height of the reservoir [13]. Ventilation followed a lung-protective strategy with low tidal volumes to avoid volume induced lung injury and sufficient PEEP to avoid alveolar collapse [13, 18]. Additionally, recruitment manoeuvres were performed at hourly intervals during EVLP (inspiratory hold on 2.5 kPa for 3 consecutive breaths). Perfusion and ventilation parameters were monitored permanently during EVLP. At the end of EVLP, the lungs were cooled to 20 °C, perfusion was stopped and the trachea was clamped at 1.5 kPa inspiratory pressure [15].

### Fixation, sampling and embedding

All lungs were perfusion fixed via the truncus pulmonalis; the lungs of the control group after explantation, the lungs of the ischaemia group after 24 h ischaemia and the lungs of both EVLP groups after 24 h ischemia and 12 h EVLP. At the time of fixation all lungs were inflated and clamped at a pressure of 1.5 kPa. To ensure that all lung regions were analysed with equal probability, an unbiased sampling cascade was applied [19]. At least 12 samples were generated per animal for light microscopy (LM) and electron microscopy (EM) by systematic uniform random sampling (SURS). SURS is recommended by the European Respiratory Society and the American Thoracic Society [20] and ensures that predilection sites for pathological alterations are neither excluded nor overrepresented. Both lungs of every animal were cut into 12 mm wide slices. From these slices, samples were excised at fixed, predefined intervals with a random start [19]. Embedding (LM: glycolmethacrylate; EM: epoxy resin) and staining procedures are described in detail in Additional file 1.

### Stereological analysis

A cascade design was employed for stereological analysis from whole lung to EM level [20]. Total lung volume as reference space was estimated by the Cavalieri Principle [21, 22] from photographs of the lung slabs using the STEPanizer® stereology tool (Version 1.1, www.stepanizer.com) [23]. LM analysis was conducted at objective magnifications 5x and 20x using a light microscope (Leica DM 6000 B, Wetzlar, Germany) and a computer-assisted stereology system (newCAST™, Visiopharm, Hoersholm, Denmark). Transmission electron microscopy was carried out using a Morgagni™ 268 (FEI, Eindhoven, Netherlands), equipped with a digital camera and a software system (Veleta and iTEM, Olympus Soft Imaging Solutions, Münster, Germany). At a magnification of 11,000x images were taken and stereological estimations were conducted with the STEPanizer®.

Volume and surface densities, absolute volumes and surfaces and bab thickness were estimated according to currently available guidelines [20]. Procedures are presented in detail in Additional file 1.

### Functional assessment

Oxygenation capacity was analysed in all groups in vivo at the time point post thoracotomy immediately before lung explantation. In aEVLP and cEVLP groups oxygenation capacity was analysed again after the lungs had been subjected to cold ischaemia and 2 and 12 h EVLP. Oxygenation was determined in blood/perfusate postlung (PaO_2_) radiometrically (ABL 700 radiometer, Willich, Germany). The oxygenation index was calculated as: OI = PaO_2_ [mm Hg]/FiO_2_ at FiO_2_ = 1 [18].

Static lung compliance (C_stat_) was analysed at the same time points as oxygenation capacity: intraoperatively, post thoracotomy immediately before lung explantation (in vivo data) in all groups and after 2 and 12 h EVLP in both EVLP groups. For determination of C_stat_, plateau airway pressure (Paw_plat_) was recorded and C_stat_ was then calculated according to the formula: C_stat_ [l/kPa] = V_t_ [l]/(Paw_plat_ [kPa] - PEEP [kPa] [18]. OI and C_stat_ data of the two EVLP groups only were published in a previous study [15] and presented in this study for comparison with new OI and C_stat_ data from control and ischaemia group.

### Statistics

IBM SPSS statistics 24 was used for statistical analysis in this explorative study. Data were tested for normality (Shapiro-Wilk test) and homogeneity of variances (Levene test). Data complying with those two prerequisites were analysed by ANOVA (analysis of variance) and post hoc Tukey Test, otherwise using the Kruskal-Wallis test including post hoc multi-comparisons with stepwise down adjustment. Repeated-measurements ANOVA was applied for functional data. Differences were considered statistically significant at *p* < 0.05.

## Results

### Ventilation-related parameters

**Fig. 1:**
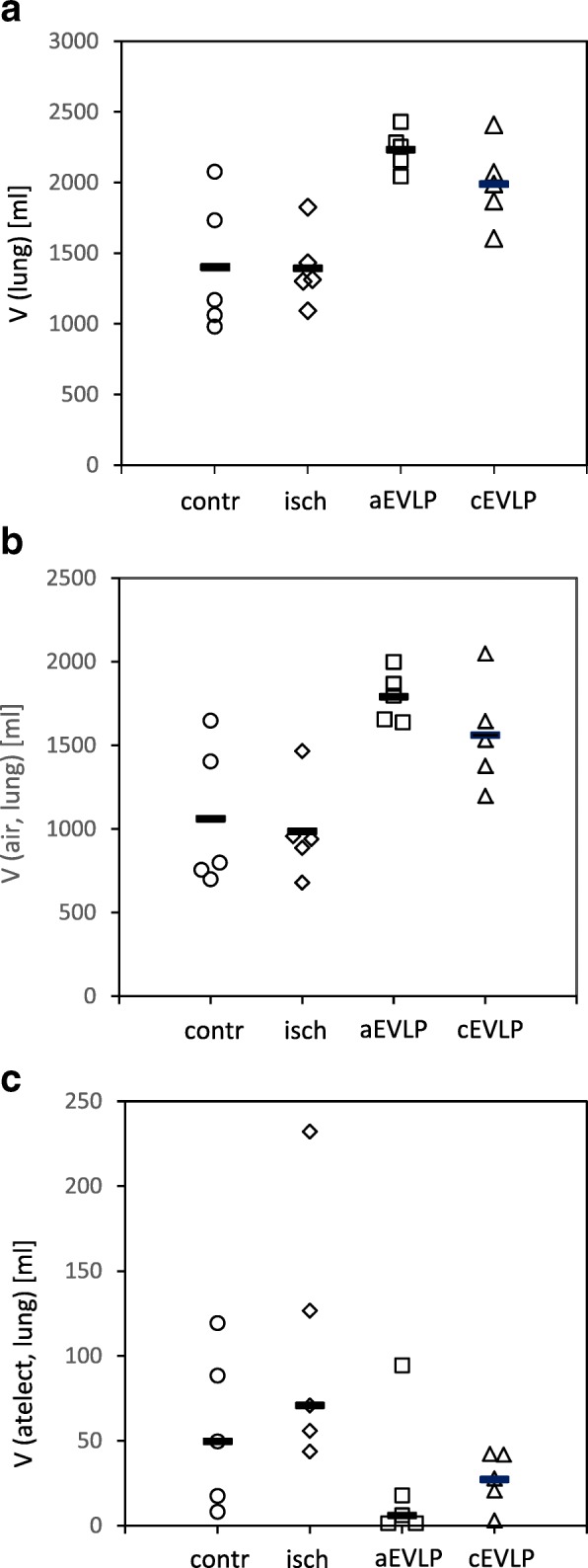
Stereological estimation of lung volume (**a**), volume of pulmonary air content (**b**) and volume of atelectatic lung parenchyma (**c**). Data points depict individual animals, bars indicate group means (**a** and **b**) or medians (**c**). Group differences were statistically significant for: (**a**) aEVLP and cEVLP vs. control group (*p* = 0.004 and *p* = 0.047, respectively) and aEVLP and cEVLP vs. ischaemia group (p = 0.004 and *p* = 0.042, respectively); (**b**) aEVLP vs. control group (*p* = 0.010) and aEVLP and cEVLP vs. ischaemia group (*p* = 0.005 and *p* = 0.049, respectively); (**c**) aEVLP and cEVLP vs. ischaemia group (both *p* = 0.048). V volume, atelect atelectatic lung parenchyma, contr control group, isch ischaemia group, aEVLP acellular EVLP group, cEVLP cellular EVLP group.

All lungs were well inflated. The volume of both lungs was significantly greater in groups aEVLP and cEVLP compared to control group (*p* = 0.004 and 0.047, respectively) and ischaemia group (*p* = 0.004 and 0.042, respectively) (Fig. 1a). Alveolar air volume differed significantly between control group and aEVLP (*p* = 0.010) and between ischaemia group and both EVLP groups (aEVLP *p* = 0.005; cEVLP *p* = 0.049) (Fig. 1b). The amount of atelectatic parenchyma (Fig. 1c) was low in all groups but higher immediately after explantation (control group mean 50 ml) and ischaemia (ischaemia group mean 71 ml) than after EVLP (group means aEVLP 6 ml and cEVLP 28 ml). Statistical significance was reached for the ischemia group versus both EVLP groups (*p* = 0.048).

All numerical data of the stereological results are found in Additional file 2: Tables S1-S3.

**Fig. 2:**
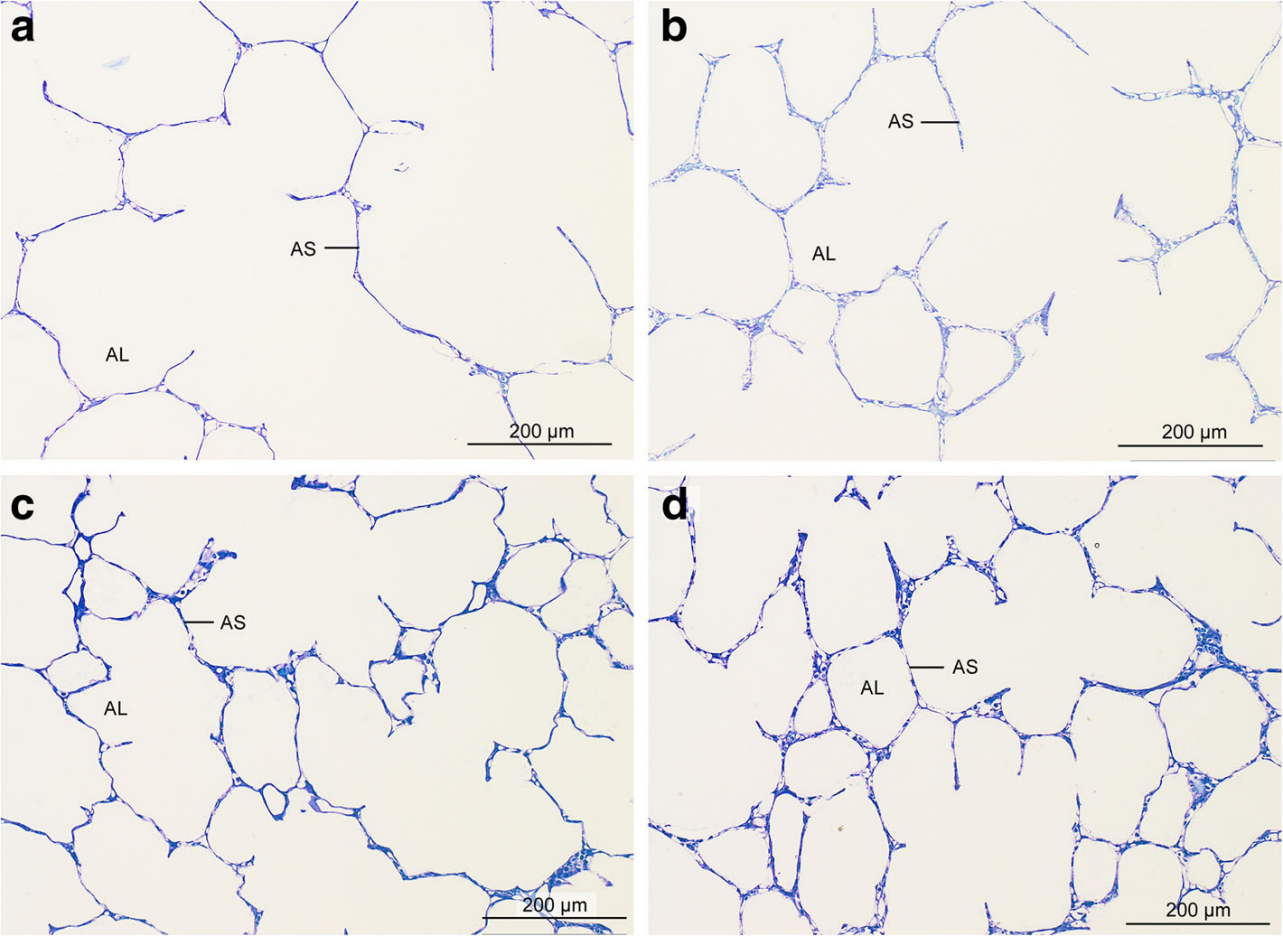
Light micrographs of lung parenchyma. Lung parenchyma was unaltered in all groups in most sections. Toluidine blue stained sections. (**a**) control group; (**b**) ischaemia group; (**c**) aEVLP group; (**d**) cEVLP group. AL air filled alveolar lumen, AS interalveolar septum

**Fig. 3:**
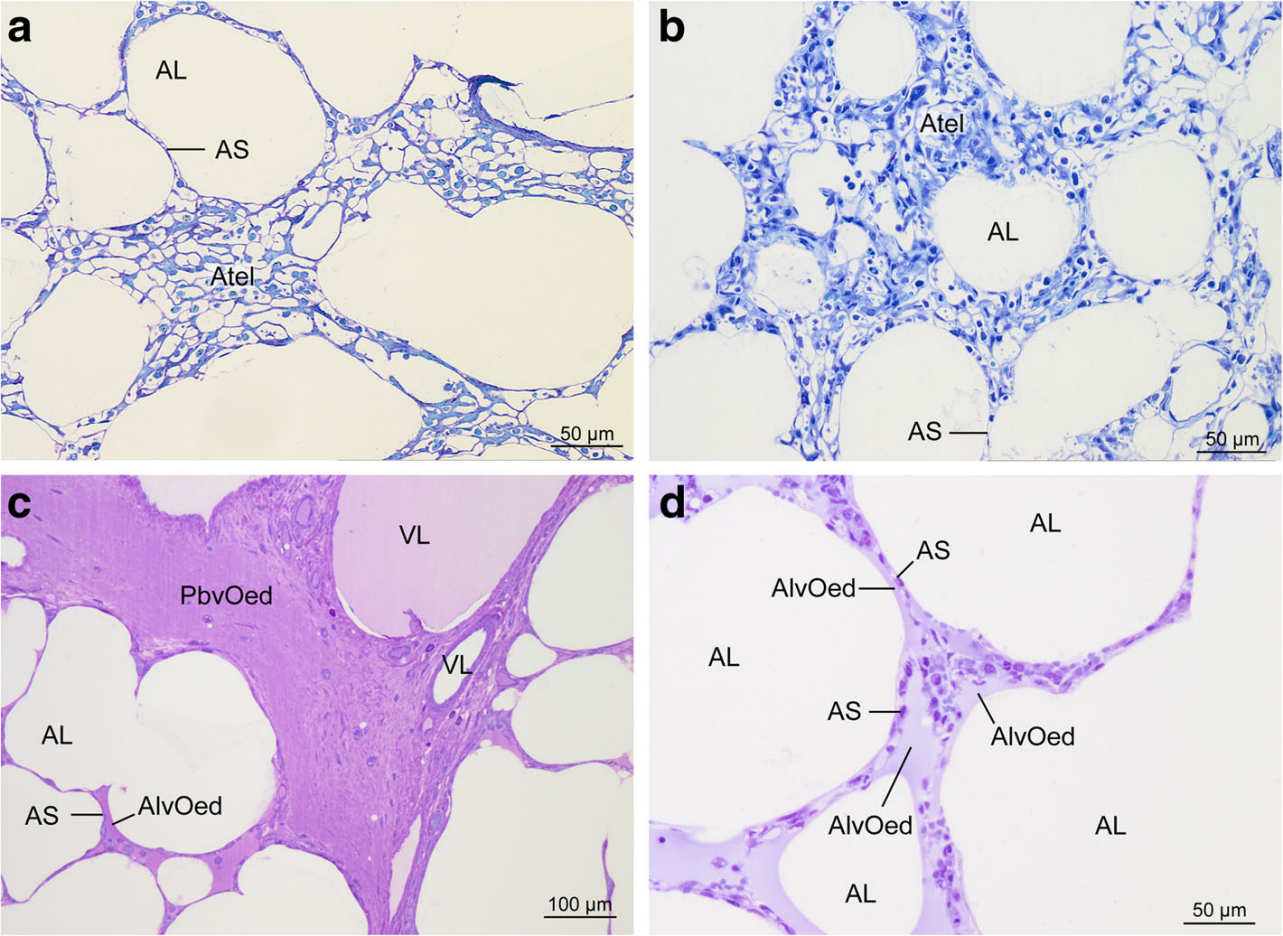
Some sections contained areas with microatelectasis or oedema formation. Microatelectasis was seen predominantly in control group (**a**) or ischaemia group (**b**). Oedema was found almost only in EVLP groups (**c**) aEVLP, (**d**) cEVLP). Light micrographs of toluidine blue stained sections. AL air filled alveolar lumen, AS interalveolar septum, Atel atelectasis, AlvOed intraalveolar oedema, PbvOed peribronchovascular oedema, VL vascular lumen

In general, histologic lung condition was excellent in all groups (Fig. 2). Lung parenchyma was unaltered in all groups in most sections and showed mostly well inflated alveoli with slender alveolar septa. Microatelectatic areas were distributed heterogeneously in all groups and were encountered only in some sections (Fig. 3). Alveolar air spaces as well as peribronchovascular connective tissue were usually free from oedema (Fig. 2) but some sections enclosed areas containing oedema fluid (Fig. 3). Oedema occurred almost exclusively in EVLP groups. It was found in the peribronchovascular compartment and/or the intraalveolar compartment in both EVLP groups.

### Alveolar septum

**Fig. 4:**
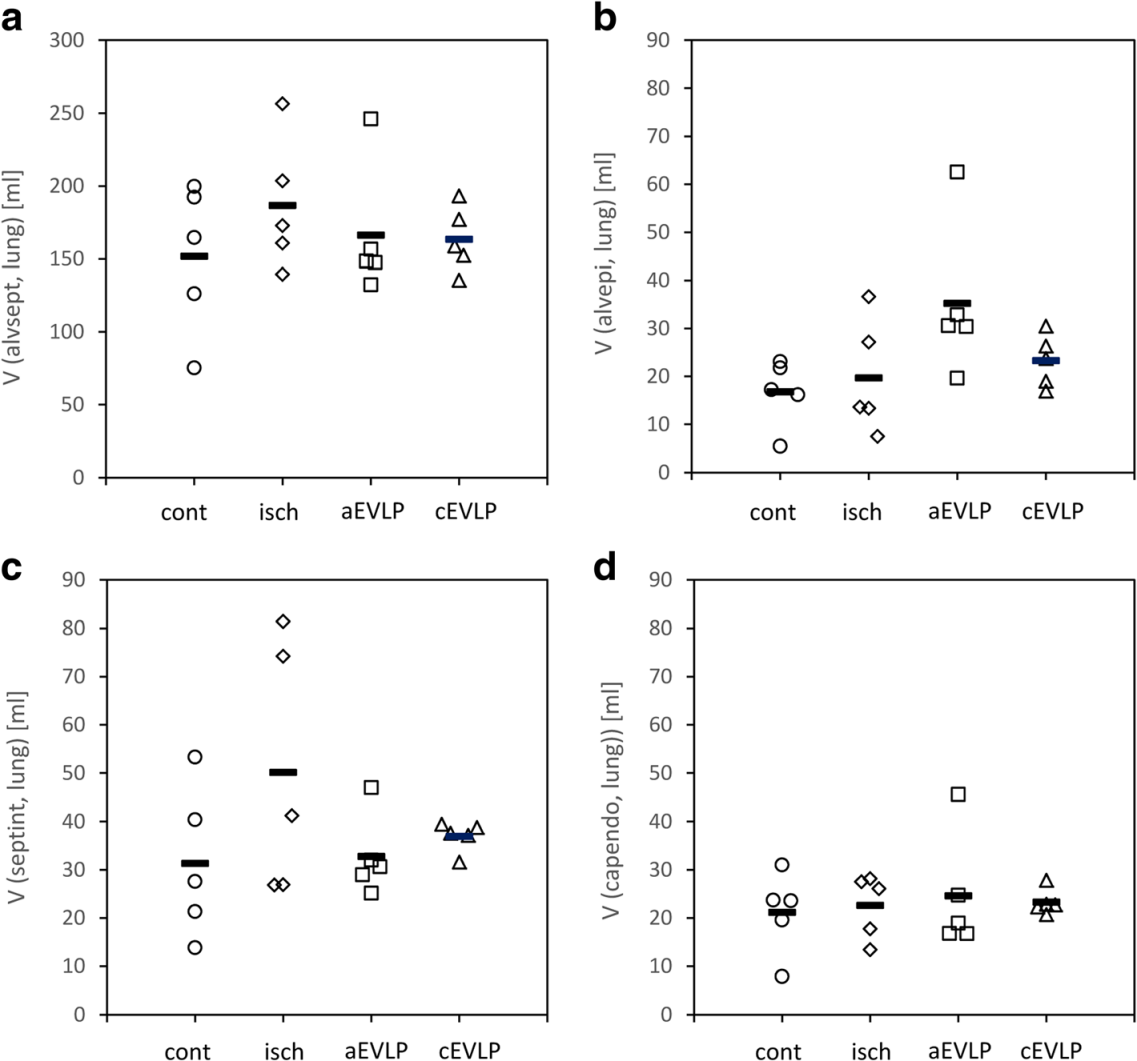
Stereological estimation of the volume of interalveolar septum (**a**), and components of the blood air barrier. Alveolar epithelium (**b**), septal interstitium (**c**) and capillary endothelium (d). None of the estimates showed significant group differences (interalveolar septum *p* = 0.636; alveolar epithelium *p* = 0.078; septal interstitium *p* = 0.262; capillary endothelium *p* = 0.930). Data points depict individual animals, bars indicate group means, V volume, alvsept interalveolar septum, alvepi alveolar epithelium, septint interstitium of interalveolar septum, capendo capillary endothelium, contr control group, isch ischaemia group, aEVLP acellular EVLP group, cEVLP cellular EVLP group

**Fig. 5:**
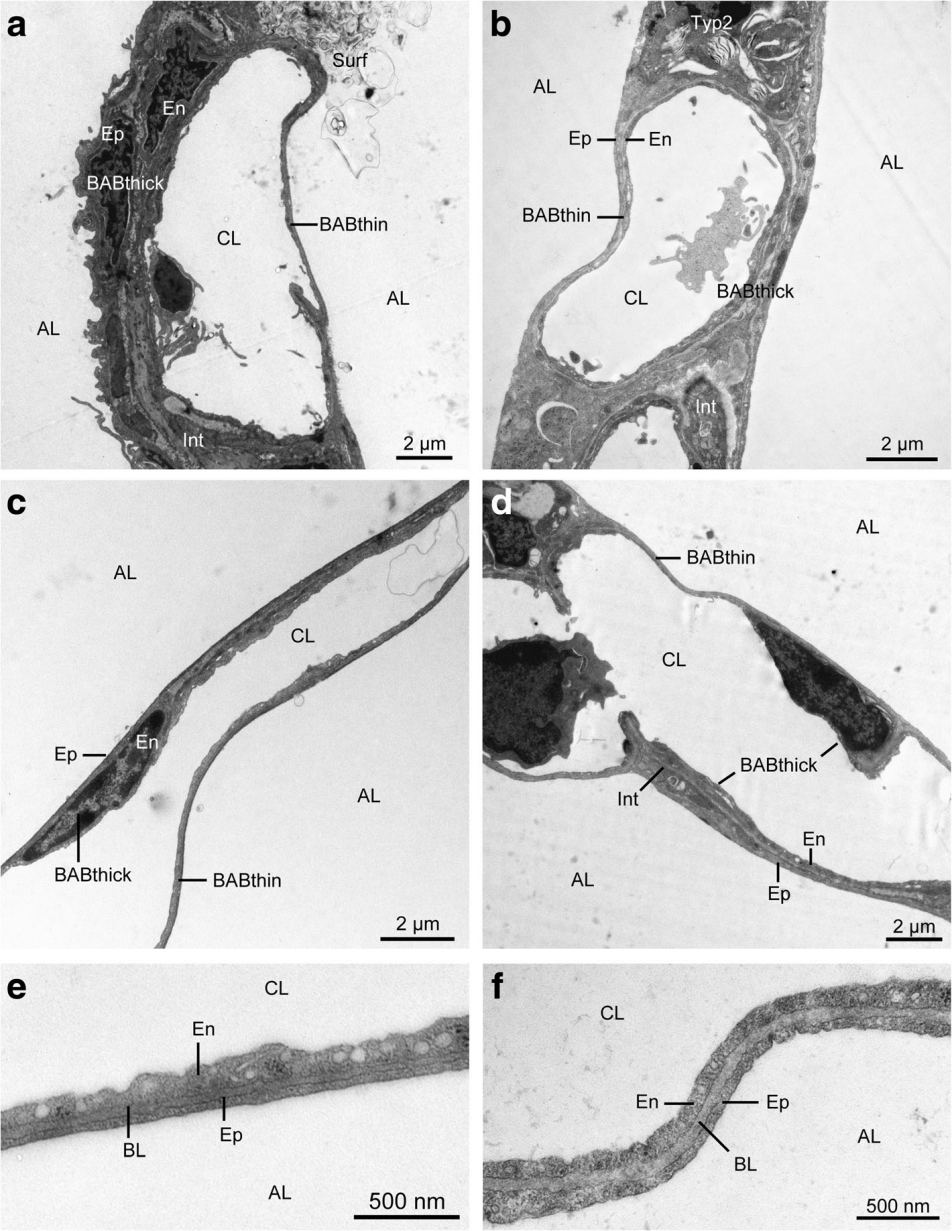
Electron micrographs of interalveolar septum. (**a**) control group; (**b**) ischaemia group; (**c**) aEVLP group; (**d**) cEVLP group. (**e**) and (**f**) High magnification of thin bab; aEVLP (**e**) and cEVLP (**f**). The alveolar septum was unaltered in most sections. AL air filled alveolar lumen, CL capillary lumen, BABthin and BABthick thin or thick part of the bab, Ep alveolar epithelium, Typ2 type 2 alveolar epithelial cell, Int septal interstitium, En capillary endothelium, NEn nucleus of endothelial cell, BL basal lamina.

The volume of alveolar septa totalled 152–187 ml (group means, Fig. 4a). Group differences were not statistically significant (*p* = 0.636). Qualitatively, tissues of bab and lumina of the septal capillary network showed a comparable ultrastructure in all 4 groups (Fig. 5). The alveolar septum was in good condition in most sections. Most alveolar septa exhibited a well perfused state as indicated by open capillary lumina. The total volume of the septal capillary network encompassed 74–94 ml (group means; Additional file 2: Table S1). The bab was continuous, without fragmentation, slender and usually without signs of oedema (Fig. 5). Alveolar epithelium, basal lamina and capillary endothelium were clearly delineated (Fig. 5e and f). The capillary endothelium volume was very homogeneous among groups (*p* = 0.930). Differences in alveolar epithelium (*p* = 0.078) and septal interstitium volumes (*p* = 0.262) were greater but did not reach significance (Fig. 4b-d).

**Fig. 6:**
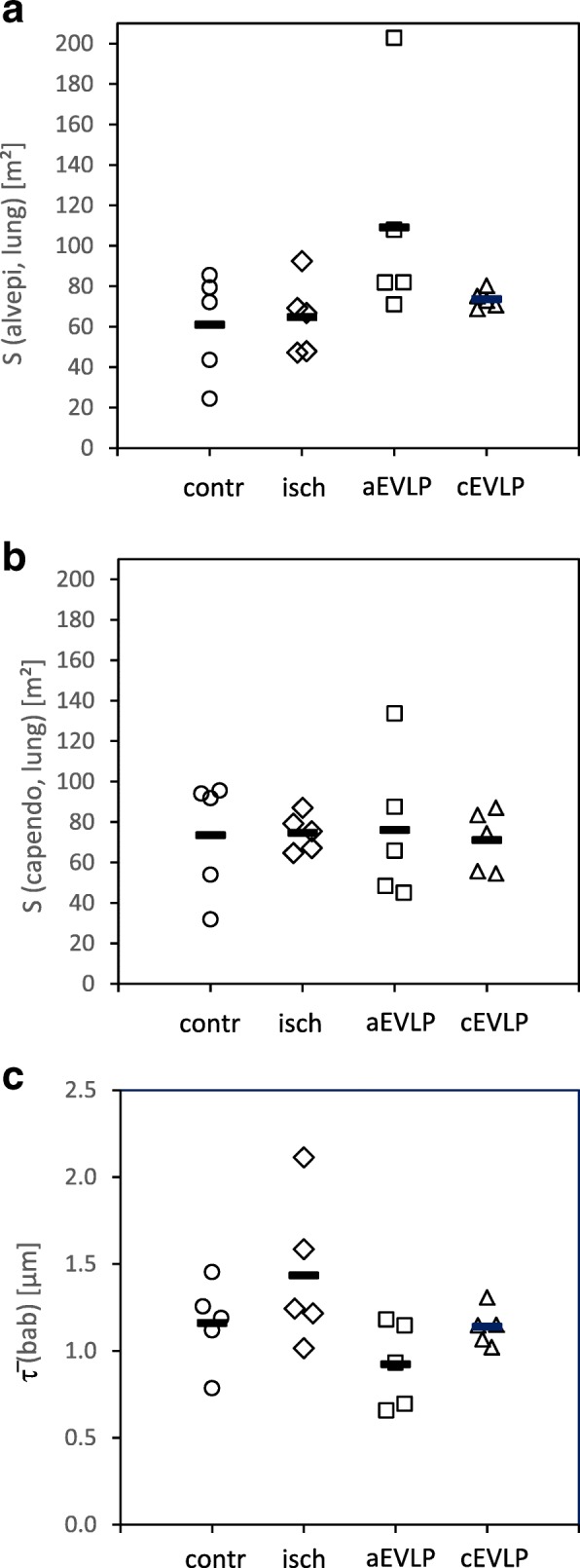
Stereological estimation of alveolar epithelial surface (**a**), endothelial surface (**b**) and blood-air barrier thickness (bab) (**c**). Group differences were not statistically significant (alveolar epithelial surface *p* = 0.103; endothelial surface *p* = 0.990; bab thickness *p* = 0.075). Data points depict individual animals, bars indicate group means, S surface area, $\overline{\uptau}$ (bab) arithmetic mean thickness of blood air barrier, contr control group, isch ischaemia group, aEVLP acellular EVLP group, cEVLP cellular EVLP group

The capillary endothelial surface was similar among groups (group means 71–76 m^2^; Fig. 6b), while the alveolar epithelial surface area showed more divergence between groups (group means 61–109 m^2^; Fig. 6a) but differences were not significant.

The arithmetic mean thickness of the bab tissues was 1.04 μm (control), 1.30 μm (ischaemia), 1.10 μm (aEVLP) and 1.17 μm (cEVLP group) (Fig. 6c). This included alveolar epithelium (group means 0.28–0.34 μm), septal interstitium (0.43–0.71 μm) and capillary endothelium (0.29–0.34 μm) (Additional file 2: Table S2). Furthermore, two conformations of the bab could be differentiated: thin and thick bab (Fig. 5). Between 31 and 46% of total alveolar surface (Additional file 2: Table S2) could be attributed to thin bab. Group assignments did not influence the thickness of total bab, its components or conformation significantly.

### Oedema related parameters

**Fig. 7:**
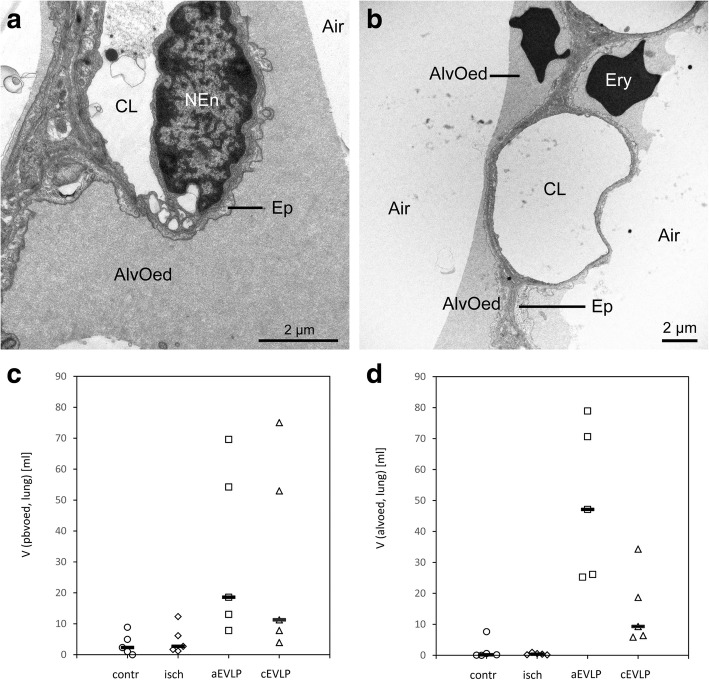
Low grade oedema formation was visible in some EM sections in both EVLP groups (**a**) aEVLP, (**b**) cEVLP). (**c**) Stereological estimation of peribronchovascular oedema. Group differences were statistically significant for aEVLP vs. control and ischaemia groups (*p* = 0.018). (**d**) Stereological estimation of intraalveolar oedema. Group differences were statistically significant for both EVLP groups compared to control and ischaemia groups and additionally in aEVLP vs. cEVLP (*p* = 0.002). Data points depict individual animals, bars indicate group medians, Air air filled alveolar lumen, AlvEd intraalveolar oedema, CL capillary lumen, Ep alveolar epithelium, NEn nucleus of endothelial cell, V volume, PbvEd peribronchovascular oedema, contr control group, isch ischaemia group, aEVLP acellular EVLP group, cEVLP cellular EVLP group

Most sections were free from oedema fluid (Figs. 2 and 5). When present, oedema was well visible in LM and EM sections (Figs. 3 and 7).

In the control and ischaemia groups, very little oedema fluid was detected and it was almost exclusively located in the peribronchovascular compartment (Fig. 7c).

After EVLP, small amounts of peribronchovascular (pbv) oedema (group medians 18.59 ml aEVLP and 11.27 ml cEVLP) and intraalveolar (alv) oedema (group medians 47.12 ml aEVLP and 9.31 ml cEVLP) were found (Fig. 7). Pbv oedema volume was significantly greater in aEVLP vs. control and ischaemia groups (*p* = 0.018). The amount of intraalveolar oedema was significantly higher in both EVLP groups compared to control and ischaemia groups and additionally in aEVLP vs. cEVLP (*p* = 0.002). Intraalveolar oedema fluid covered the alveolar epithelium and accumulated in particular in alveolar niches. In these areas, alveolar epithelium also showed oedematous swelling (Fig. 7). 27.98 and 4.98 m^2^ of alveolar surface were covered with a fluid film of 2.66 and 2.75 μm thickness in aEVLP and cEVLP, respectively (group medians; Additional file 2: Table S3). Statistical significance was reached for alv oedema surface (both EVLP groups vs. control and ischaemia groups and aEVLP vs. cEVLP; *p* = 0.001). Thin and thick bab parts were affected equally (Additional file 2: Table S3). Oedema fluid was distributed very heterogeneously within and between animals.

### Functional data

Oxygenation was well within the physiological range at all time points and in all groups (Table 1). OI did not differ significantly between groups (*p* = 0.912) or time points (*p* = 0.557). Intraoperative OI was determined in all groups and ranged between 469 and 520 (group means; Table 1). In EVLP groups, OI remained on in vivo levels until the end of the experiment (12 h EVLP, group means aEVLP 496, cEVLP 514). Static lung compliance did not show significant group differences (*p* = 0.549). In the EVLP groups, C_stat_ differed significantly between time points (*p* = 0.004). In both EVLP groups, it was greater after 2 h EVLP than in vivo and decreased towards the end of EVLP (Table 1). OI and C_stat_ data of the two EVLP groups were taken from Becker et al. [15].

**Table 1:** Oxygenation index and static compliance in vivo and during EVLP

Time point	Group	OI	C_stat_ [l/kPa]
In vivo	Control	520 ± 91	0.66 ± 0.17
	Ischaemia	487 ± 87	0.52 ± 0.14
	aEVLP	496 ± 64	0.65 ± 0.10
	cEVLP	469 ± 10	0.64 ± 0.19
2 h EVLP	aEVLP	482 ± 41	0.86 ± 0.12
	cEVLP	506 ± 55	0.76 ± 0.27
12 h EVLP	aEVLP	496 ± 41	0.57 ± 0.08
	cEVLP	514 ± 61	0.55 ± 0.12

Mean ± SD. In vivo data were obtained post thoracotomy immediately before lung explantation. Data of 2 and 12 h EVLP were obtained after 24 h cold ischemia and 2 and 12 h EVLP, respectively. Data of aEVLP and cEVLP groups were taken from [15]. OI did not differ significantly between groups (*p* = 0.912) or time points (*p* = 0.557). C_stat_ was not significantly different between groups (*p* = 0.549) but differed significantly between all 3 time points for EVLP groups (*p* = 0.004). OI oxygenation index at FiO_2_ = 1, C_stat_ static lung compliance

## Discussion

In this large animal ex vivo ischaemia and ex vivo reperfusion model, we compared prolonged aEVLP and cEVLP. A previous study had demonstrated higher PAP and PVR in cEVLP but the impact of this finding remained unclear [15]. The objective of this study was to identify which EVLP technique is better suited to sustain lung cells, functional lung structure and to prevent IER injury development. The cellular relevance of these findings was elucidated by quantitative structural and ultrastructural analysis and a hypothesis was generated regarding possible underlying mechanisms.

### EVLP model

Surgical procedures in this porcine model were performed comparable to donor lung procurement in a clinical context [24]. After recovery, the organs (except those of the control group) were subjected to 24 h cold ischaemia to impose well defined stress on the lungs. This time period exceeded accepted ischaemic times in clinical lung transplantation considerably [2]. Ischaemic times greater than 6–8 h were associated with an increased propensity of IR injury, PGD and inferior transplantation outcome [2, 25, 26]. Several experimental studies demonstrated that 18 to 24 h of cold ischaemia is an intensive stressor that leads to severe IR injury and impairment of lung function upon reperfusion [6, 27, 28].

In clinical EVLP different protocols are commonly followed. They differ in several aspects, mainly in the composition of perfusion solutions, EVLP circuit set-up, and perfusion flow (i.e. 40 or 70 ml/kg bw/min) and pressures [11, 14, 29]. The protocol of our study was developed to differ solely in the addition of erythrocytes to the perfusion solution in order to differentiate the impact of erythrocyte enrichment from other perfusion parameters in this prolonged setting of 12 h EVLP. Perfusion times in clinical EVLP range from 4 to 11 h. For functional assessment of lungs after donation after circulatory death (DCD) or of marginal lungs after donation after brain death usually shorter time periods are used, while for reconditioning purposes or therapeutic interventions an extension of perfusion times up to 6–12 h or even beyond might be necessary [30–32]. In experimental EVLP, perfusion times of up to 24 h have been performed [17, 33].

Resolving the controversy regarding optimal perfusion strategies in prolonged EVLP could promote EVLP application and thus contribute to the alleviation of the donor shortage in lung transplantation.

### Effect of EVLP after cold ischaemic challenge

Lung samples of our four experimental groups were studied stereologically with respect to morphological alterations due to ischaemia and EVLP. In particular, parameters related to lung function and IER injury were analysed. IR injury/IER injury is a complex entity that is encountered when organs are deprived from vascular perfusion for a certain time period (ischaemia) and perfusion is resumed afterwards [34]. Upon explantation from the donor, an ischaemic phase is imposed on all lung grafts. In lung transplantation, reperfusion is initiated in vivo after connecting the vasculature of the transplanted organ to recipient circulation [35]. All lungs subjected to an ischaemia and reperfusion sequence will experience organ stress and detectable organ injury is recognized in vivo as IR injury [3, 6]. Reperfusion can also be performed in ex vivo systems, in particular for experimental purposes [4, 5, 36–38]. In vivo and ex vivo reperfusion differ in particular with respect to systemic effects, e.g. like influx of neutrophils and lymphocytes from systemic recipient circulation [39] which has to be taken into account when interpreting ex vivo findings. The pathogenesis of IR injury/IER injury is characterized by a multitude of morphological, molecular, biochemical, immunological and functional alterations and has been studied intensively but is still not fully understood [4, 5, 34, 37, 39]. Severe forms of IR injury/IER injury can result in acute organ dysfunction. The clinical condition was defined as Primary Graft Dysfunction (PGD) by the International Society for Heart and Lung Transplantation and graded based on impaired oxygenation capacity and radiographically detectable oedema formation [40]. However, models leading to less severe forms of IR injury/IER injury are particularly suited to bring light to the sequence of events and underlying mechanisms during IR injury/IER injury development. The ex vivo lung perfusion (EVLP) system is used for clinical and experimental reasons and allows functional assessment, reconditioning and interventional procedures during an ex vivo reperfusion phase [7, 10, 14]. If lungs are to be transplanted into a recipient after EVLP, a second ischaemic phase is inflicted onto the lungs, which lasts from disconnecting the organ from the EVLP circuit until a connection with recipient vasculature is established and which is followed by the second, in vivo, reperfusion phase [11, 30]. In our study, the ischaemic insult was represented by a 24 h period of cold ischaemia. Reperfusion was performed ex vivo in the EVLP system. The aim of our study was to examine the effect of EVLP on the condition of the lung graft. Therefore, implantation into a recipient with its second ischaemia and second reperfusion phase was not carried out.

In our study, no significant morphological damage was visible after cold ischaemia alone. This corresponds to previous findings in ischaemia induced lung injury which did not manifest until the reperfusion phase [5, 27, 28, 41]. In these studies, only groups also subjected to reperfusion developed marked pulmonary injury like cell death, bab disintegration and severe lung dysfunction [5, 41].

In both EVLP groups, lungs were reperfused ex vivo for 12 h following ischaemia. After altogether 36 h ex vivo lungs remained in good condition in both EVLP groups according to qualitative and quantitative LM and EM analysis as well as functional findings.

Parenchymal ventilation was even improved after EVLP (Fig. 1). During explantation surgery, a recruitment manoeuvre was performed in order to minimize the amount of atelectasis formation. However, formation of atelectasis could not be prevented completely and a small amount of atelectatic tissues was detected in control and ischaemia groups. During EVLP, lungs were ventilated continuously in a lung protective mode and recruitment manoeuvres were performed at hourly intervals in both EVLP groups. The volume of atelectatic lung parenchyma was reduced significantly in both EVLP groups compared to ischaemia group which demonstrated that the ventilation strategy was beneficial and that the small amount of atelectasis formed during explantation and storage was largely reversible. Significant differences between aEVLP and cEVLP were not noted for ventilation related parameters. Good alveolar ventilation and avoidance of atelectasis is essential for preservation of alveolar septum integrity and function during EVLP since the septal oxygen supply is primarily derived from the alveolar air space [24, 42].

The condition of the alveolar septum is of particular importance during lung transplantation. A good condition at the time of implantation is a prerequisite for sustained organ performance in the immediate post-transplant period as well as for long-term success [2, 24, 43]. In our model, alveolar septum integrity was preserved well in all groups. Quantification of its components provided the basis for assessing bab thickness, diffusion capacity and septal oedema formation [20, 44]. Total thickness of porcine bab in our study equalled thickness in weaner pigs (28–32 kg body weight) (1.06 μm) [45], but was slightly thinner than in newborn (1.65 μm) and 30 d old piglets (1.55 μm) [46]. Human bab was thicker (2.2 μm) [44], but the bab of small rodents much thinner (0.36 μm) [4]. In our study, none of the quantitative measures of the bab and its components exhibited significant group differences, indicating that the structural basis for physiological gas exchange was preserved in both EVLP groups. With regard to IR injury/IER injury development, mainly two structural aspects have to be considered: injury of bab components and oedema formation. The bab showed no signs of IER injury in our study.

Oedema formation is the key event during manifestation of IR injury/IER injury [5]. Radiographically visible oedema belongs to the compulsory features determining diagnosis of PGD [40]. During oedema formation, a typical sequence of events can be observed: fluid accumulation usually starts in the peribronchovascular compartment, then extends into alveolar septa and further aggravation leads to transgression into intraalveolar air space [47]. In both EVLP groups, almost similar, small amounts of were present (Fig. 7c) indicating low level IER injury.

Septal oedema leads to an increase in bab thickness which is inversely proportional to diffusion capacity [42]. In rat IR injury studies, septal oedema was mainly localized in septal interstitium [4]. In our study, no quantitative indication of septal oedema formation was found in any of the groups as determined by volume and thickness of total bab or its individual components (alveolar epithelium, septal interstitium, capillary endothelium) (Figs. 4 and 6).

Intraalveolar oedema is functionally the most relevant form. A volume fraction of more than 3% alveolar oedema compromised oxygenation in a rat model [4, 48]. Small amounts of alveolar oedema were found in both EVLP groups (Fig. 6d) but remained below the critical volume fraction of 3% in both EVLP groups. An impairment of oxygenation towards the end of EVLP occurred neither in aEVLP nor in cEVLP in our model, thus functional and morphological findings correlated well.

Signs of IER injury were only encountered in oedema parameters but not in bab parameters or oxygenation. Additionally, the amount of peribronchovascular and intraalveolar oedema was small indicating that only low grade IER injury had developed in both EVLP groups. Thus, development of substantial and functionally relevant lung injury was prevented even though the lungs had been subjected to 24 h ischaemia and spent in total 36 h ex vivo. Despite that the amount of intraalveolar oedema was low in both EVLP groups, it has to be noted that intraalveolar oedema volume was statistically significantly higher in aEVLP than in cEVLP (group medians 47 vs. 9 ml). We generated a hypothesis that the difference in oedema volume between aEVLP and cEVLP could be due to slightly inferior perfusion of the interalveolar septum in aEVLP compared to cEVLP (Table 2). Septal perfusion requires capillary pressure exceeding alveolar air pressure. In a physiological in vivo situation (spontaneous breathing, closed thorax) septal capillary pressure [42] exceeds alveolar air pressure [49] during inspiration (+ 0.85 kPa) and expiration (+ 0.65 kPa) and the interalveolar septum can be perfused well during the complete respiratory cycle (Table 2). During EVLP, the situation is more complex. In a previous study of our group, which focused on lung physiology during EVLP, perfusate pressure and airway pressure data were recorded during EVLP [15] and these values are listed in Table 2. PAP was significantly higher in cEVLP than in aEVLP [15]. This was probably due to a higher viscosity of the erythrocyte containing perfusate, which increased pulmonary vascular resistance and, at equal perfusion flow, resulted in higher vascular pressures [50–53]. As a consequence of higher PAP, estimated capillary pressure was higher in cEVLP than in aEVLP (estimated values after 12 h EVLP: aEVLP 0.78 kPa, cEVLP 1.10 kPa, Table 2). In both EVLP groups, estimated capillary pressure was less than alveolar air pressure during inflation (aEVLP -0.48 kPa, cEVLP -0.10 kPa, Table 2) probably resulting in compression of septal capillaries and perfusion stop. Thus, we assume that the majority of interalveolar septa was not perfused during inflation in both EVLP groups. During deflation, estimated capillary pressure exceeded alveolar air pressure in both EVLP groups (aEVLP 0.28 kPa, cEVLP 0.60 kPa, Table 2), thus probably allowing septal perfusion during this phase of the respiratory cycle. The magnitude of pressure difference during deflation was comparable to a physiological situation in cEVLP (Table 2). The difference was lower in aEVLP and possibly resulted in inferior septal perfusion in this group. We hypothesize that inferior perfusion of the interalveolar septum might compromise bab metabolism and lead to light cellular injury. We found swelling of alveolar epithelium in oedema covered areas as a morphological correlative. In this way alveolar clearance could be reduced and result in increased intraalveolar oedema formation in aEVLP compared to cEVLP.

**Table 2:** Estimated perfusion of the interalveolar septum in physiological conditions and in EVLP

	Physiological^a^	aEVLP	cEVLP
Blood/perfusate pressures			
PAP [kPa]	1.13^b^	1.27^c^	1.89^c^
Capillary pressure [kPa]^d^	0.75	0.78	1.10
LA pressure [kPa]	0.38^b^	0.30^c^	0.30^c^
Alveolar air pressures			
Inspiratory pressure/ Plateau airway pressure [kPa]	− 0.10^e^	1.26^c^	1.24^c^
Expiratory pressure/ PEEP [kPa]	0.10^e^	0.50^c^	0.50^c^
Blood/perfusate - alveolar air pressure difference			
Inspiration/inflation: Capillary pressure - Inspiratory pressure/ Plateau airway pressure [kPa]	0.85	−0.48	−0.14
Expiration/deflation: Capillary pressure - Expiratory pressure/ PEEP [kPa}	0.65	0.28	0.60

Perfusion of the interalveolar septum depends on blood/perfusate pressure in septal capillaries and alveolar air pressure. When capillary pressure exceeds alveolar air pressure (positive difference) septal capillaries can be perfused. When the difference is negative, capillaries will be compressed and perfusion cannot be sustained

^a^Pressures and pressure relationships in a physiological in vivo situation (spontaneous breathing, closed thorax); ^b^From Ochs and O’Brodovich [42]; ^c^From Becker et al. [15], after 12 h EVLP; ^d^Lung capillary pressures are approximately halfway between pulmonary arterial and pulmonary venous pressures [42]; ^e^ from Kunzelmann and Thews [49]. PAP mean pulmonary artery pressure, LA left atrium, PEEP positive end-expiratory pressure

Moreover, estimated values for pressure differences describe a mean perfusion situation. However, intrapulmonary variations in lung perfusion pressures exist due to gravitational forces and regional blood flow regulation [49]. These variations are likely to facilitate/impede local septal perfusion, in particular when capillary-alveolar air pressure differences are marginal. Thus perfusion deficiencies would not affect all alveolar septa equally. Our observation that alveolar oedema was distributed very heterogeneously within the lungs corroborates this idea. Since in aEVLP the overall pressure situation is less favourable and assuming normal intrapulmonary pressure variability, more alveolar septa might experience impaired perfusion. The morphological equivalent is a larger oedema covered alveolar surface area compared to cEVLP. Alltogether, we deduct that the main effect of erythrocyte addition to perfusate is its impact on capillary pressure which, in turn, is responsible for the differences we found between aEVLP and cEVLP. Further studies focusing on septal perfusion and quantitative cellular metabolism are needed to confirm this hypothesis.

The assumption that a less favourable relationship between capillary pressure and alveolar air pressure could be the reason for higher oedema volumes in aEVLP was corroborated by results of Aboelnazar et al. In their study, negative pressure ventilation decreased lung oedema in porcine EVLP [54]. Negative pressure ventilation would increase the capillary pressure – alveolar air pressure difference at a given perfusion pressure. Another recent study [14] compared acellular and cellular EVLP also in a porcine model (2 h ischaemia at 8 °C, 4 h EVLP, perfusion flow 40 ml/kg). In that study, substantial oedema formation occurred in both groups but more pronounced and in part severe in the acellular group. Possibly, in our study, higher perfusion flow (70 ml/kg/min) and thus better perfusion of the alveolar septum largely prevented IER injury development.

On the other hand, high perfusion pressures can cause hydrostatic oedema and ventilation pressures must be high enough to avoid alveolar collapse [13]. Thus, the margins regarding pressure relationships are quite narrow in EVLP.

### Strength and limitations

Strengths of our study include the animal model, EVLP protocol and stereological analysis. In order to mimic human dimensions as closely as possible, we used quite large pigs in our study. The animals had a mean body weight of 57 kg and size related respiratory and cardiovascular parameters (volumes, pressures) including respective ex vivo parameters. We used an animal model with stringently controlled experimental conditions. All lungs of ischaemia group and EVLP groups were subjected to a well-defined, identical stressor. Intraoperative ventilation parameters (all groups), perfusion and ventilation parameters during EVLP (EVLP groups) and parameters during fixation (all groups) were set at defined, identical values for all animals and controlled permanently during the experiment. As lungs are very sensitive to pressure and volume variations, these strictly controlled conditions are of particular importance in lung stereology and to facilitate the identification of cause-effect relationships.

A further strength of our study was the development of a protocol for the two EVLP regimens that differed only in one factor, i.e. addition of erythrocytes, which allowed attributing group differences to this single factor and its consequences.

Our study was the first analysing lung structure and ultrastructure stereologically in a large animal EVLP model. Design-based stereology provides the tools for a quantitative analysis from whole lung to cellular and subcellular level that is representative for the entire organ [20, 55, 56]. This is of particular advantage when lesions are distributed inhomogenously within the organ, like e.g. oedema. Additionally, not only ratios but also absolute quantities of structures and lesions in the lung can be and were determined by stereology. Absolute data should be preferred in the lung whenever possible [20, 55, 57] since ratios of tissue components and lesions are greatly influenced by ventilation. Through stereology we were able to generate unbiased, detailed morphological results, compare them to functional findings and in this way elucidate structure-function relationships.

However, our study also inherits some limitations. The results were obtained in ex vivo animal experiments and cannot be extrapolated directly to clinics. Our model subjected the lungs to 24 h of cold ischaemia, a classical stressor for IR injury/IER injury development [4, 36, 58]. In a clinical setting, donor lungs are often subjected to very heterogeneous forms of damage. This can include but is not restricted to hyperinfusion, ventilator-induced lung injury, aspiration, thrombosis, infection, contusion, cytokine storm during brain death or warm ischaemia with or without ventilation in DCD lungs as well as pre-existing chronic lung damage from smoking or environmental exposures [2, 30, 59]. Each aetiology of lung injury might cause its specific forms of functional or morphological damage which might differ from classical, cold ischaemia induced injury and could contribute to differential organ performance during EVLP. A porcine warm ischaemia model demonstrated that already 1 h warm ischaemia without ventilation and 2 h with or without ventilation resulted in pulmonary oedema formation and inferior oxygenation after 8 and 24 h EVLP compared to controls [33].

The reperfusion phase was conducted ex vivo using the EVLP system in our experimental model. In an ex vivo environment, systemic recipient responses cannot be modelled. This is of particular importance with regard to IR injury development. In vivo, IR injury pathogenesis incorporates resident donor lung factors (e.g. resident cell injury, cell death, molecular changes, alveolar macrophage activation) and also systemic recipient factors (e.g. influx and activation of resident neutrophils and lymphocytes) [3, 34, 37, 39]. To differentiate between the in vivo and ex vivo response, the term “ischaemia ex vivo reperfusion associated lung injury (IER injury)” was introduced to describe the findings in our ex vivo study.

Additionally, species and age differences might exist which prohibit direct translation of results from animal experiments to a clinical setting.

Furthermore, stereology in LM and EM analysis is a material consuming technique [56]. It is not possible to sample the same organ repeatedly, for example after EVLP and after implantation into a recipient. The aim of our study was to elucidate the impact of two different EVLP regimens on lung condition. Therefore we chose the time point “after EVLP” for analysis. EVLP was developed to assess lungs ex vivo and to predict their suitability for implantation from ex vivo functional findings. The approach has been applied successfully in experimental as well as clinical situations [7, 10, 11, 30]. However, ex vivo ventilation and ex vivo reperfusion during EVLP differs from in vivo ventilation/reperfusion after implantation and post-transplant outcome cannot be linked to performance during EVLP under all circumstances. Therefore, it cannot be excluded that the lungs of our study would have performed differently in vivo after implantation. Additionally, because of its tissue consuming nature, it is not possible in clinical sampling to conduct a complete stereological analysis comparable to the methodology of our study.

## Conclusions

Both EVLP protocols supported the lungs well. After heavy challenge of 24 h cold ischaemia, both acellular and cellular EVLP preserved ultrastructural integrity well in our prolonged 12 h ex vivo reperfusion setting. Thus, both protocols can be applied with benefit. In our setting, cEVLP presented with slightly superior results regarding intraalveolar oedema formation. We generated a hypothesis on the underlying mechanism: cellular perfusate increased septal capillary pressure, improved septal perfusion and in this way preserved cellular function better. Moreover, the hypothesis emphasized that the relationship between perfusion pressure and ventilation pressures is very delicate at alveolar level in EVLP. Today’s EVLP protocols are likely to support septal perfusion in large parts of the lung only during the deflation phase.

## Additional files

Additional file 1: Detailed description of procedures for fixation, sampling and embedding and Detailed description of procedures for stereological analysis. (DOCX 35 kb)

Additional file 2:**Table S1.** Stereological data on lung structure and alveolar septum composition: absolute volumes and volume densities. Mean ± standard deviation except for ^1^. Group differences were tested for statistical significance by ANOVA and post hoc Tukey test except for ^1^. ^1^ Data deviated from normal distribution and/or homogeneity of variances; thus median ± interquartile range are listed and the Kruskal-Wallis test was used for analysis of group differences. Significance of differences (*p* < 0.05) is indicated by superscript letters. Groups marked with the same letter do not differ significantly. V absolute volume, V_V_ volume density, npar non-parenchyma, par parenchyma, air alveolar air space, atelect atelectasis, alvsept interalveolar septa, surf surfactant, alvepi alveolar epithelium, septint septal interstitium, capendo capillary endothelium, caplum capillary lumen, bab blood-air-barrier. **Table S2.** Stereological data on alveolar and capillary surface areas and thickness of blood-air barrier components. Data are given as mean ± SD. Group differences were tested for statistical significance by ANOVA and post hoc Tukey test. Significance of differences (*p* < 0.05) is indicated by superscripts. Groups marked with the same letter do not differ significantly. S surface area, S_V_ surface density, S_S_ relative surface area, $\overline{\uptau}$ arithmetic mean thickness, alvepi alveolar epithelium, capendo capillary endothelium, alvepi thin alveolar epithelium of thin bab, bab blood-air barrier, septint septal interstitium, thin bab thin part of bab, thick bab thick part of blood-air barrier. **Table S3.** Stereological data on oedema parameters. Data are given as median ± interquartile range. Group differences were tested for statistical significance using the Kruskal-Wallis test. Significance of differences (*p* < 0.05) is indicated by superscripts. Groups marked with the same letter do not differ significantly. V volume, V_V_ volume density, S surface area, S_S_ relative surface area, $\overline{\uptau}$ arithmetic mean thickness, pbv ed. peribronchovascular edema, alv oed alveolar oedema, ed-alvepi alveolar epithelial surface covered with oedema fluid, oed thin alveolar epithelium of the thin blood-air barrier (bab) covered with oedema fluid, alvepi thin alveolar epithelium of thin bab, oed thick alveolar epithelium of the thick bab covered with oedema fluid, alvepi thick alveolar epithelium of thick bab. (DOCX 37 kb)

## Abbreviations

aEVLP: Acellular ex vivo lung perfusion
alv: Intraalveolar
ANOVA: Analysis of variance
bab: Blood-air barrier
bw: Body weight
cEVLP: Cellular ex vivo lung perfusion
DCD: Donation after circulatory death
EM: Electron microscopy
EVLP: Ex vivo lung perfusion
FiO_2_: Fraction of inspired oxygen
IER injury: Ischaemia ex vivo reperfusion associated lung injury
IR injury: Ischaemia reperfusion injury
LA: Left atrium
LM: Light microscopy
PAP: Pulmonary artery pressure
pbv: Peribronchovascular
PEEP: Positive end-expiratory pressure
PGD: Primary graft dysfunction
