# Supplementary material for: Cellular and acellular ex vivo lung perfusion preserve functional lung ultrastructure in a large animal model: a stereological study
Source: Respir Res. 2018 Dec 4;19:238. doi: 10.1186/s12931-018-0942-5 (PMC6278069; doi:10.1186/s12931-018-0942-5)
Supplement: Supplementary file 1 — Detailed description of procedures for fixation, sampling and embedding and Detailed description of procedures for stereological analysis. (DOCX 35 kb) [file 12931_2018_942_MOESM1_ESM.docx]

**Additional file 1:**

**Detailed description of procedures for fixation, sampling and embedding**

Lungs were perfusion fixed through the truncus pulmonalis with 4 l cold 1.5% glutaraldehyde/1.5% paraformaldehyde solution at a constant hydrostatic pressure of 3 kPa. The trachea remained clamped during fixation.

To ensure that all regions were analyzed with equal probability, an unbiased sampling cascade was applied [1]. Lungs were embedded in agar (Carl Roth, Karlsruhe, Germany) and cut into 12.9 mm thick slabs with a tissue slicer specifically designed for porcine lungs [2]. Using a point grid, systematic uniform random sampling was employed to generate lung samples for light microscopy (LM) and electron microscopy (EM). Per animal at least 12 samples were excised for LM and EM.

All specimens were post-fixed in OsO_4_ and stained *en bloc* with uranyl acetate [3;4]. LM samples were embedded in glycolmethacrylate (Technovit 8100®, Heraeus Kulzer, Wehrheim, Germany) and sections were stained with toluidine blue. For transmission electron microscopy, samples were embedded in epoxy resin (Glycid ether 100, Serva, Heidelberg, Germany) and ultrathin sections were stained with lead citrate and uranyl acetate [3].

**Detailed description of procedures for stereological analysis**

All lungs were subjected to a cascade sampling design: Level 1: whole lung, level 2: LM sections, level 3: EM sections. Pictures were taken at each level.

Volume densities (V_V_) and absolute volumes (V) of the following lung structures were estimated: lung non-parenchyma, lung parenchyma, alveolar air space, atelectasis, surfactant, interalveolar septum, blood-air barrier (bab), alveolar epithelium, septal interstitium, capillary endothelium, capillary lumina, peribronchovascular oedema, intraalveolar oedema. For estimation of V_V_, a point grid was superimposed onto the pictures and points (P) hitting structures of interest (struct) and reference space (ref) were counted. V_V_ and V of each parameter was calculated according to equations 1a and 1b [1;5].

$V_{V}= \frac{P(struct)}{P(ref)}$ (1a)

$V\left( struct, ref \right)=V_{V}\left( struct/ref \right) \cdot V(ref)$ (1b)

Surface densities (S_V_) and absolute surface (S) of alveolar epithelium and capillary endothelium were estimated with the aid of a combined point and line grid which was superimposed onto the electron micrographs. Intersections of test lines (I) with structures as well as points hitting structures of interest were counted. S_V_ and S were then calculated according to equations 2a and 2b [1;5].

$S_{V} \left( struct/ref \right)=\frac{2 \cdot\sum I(struct)}{l\left( p \right) \cdot\sum P(ref)}$ (2a)

$S \left( struct, ref \right)=S_{V} \left( struct/ref \right) \cdot V(ref)$ (2b)

l(p): length of the test line per test point

Arithmetic mean thickness ($\overline{}$) of the bab and its components were calculated according to equation 3 [1;6;7].

$\overline{} =\frac{V_{V(struct/ref)}}{S_{V}(struct/ref)} \cdot$2 (3)

References

1. Hsia CC, Hyde DM, Ochs M, Weibel ER. An official research policy statement of the American Thoracic Society/European Respiratory Society: standards for quantitative assessment of lung structure. Am J Respir Crit Care Med. 2010;181:394-418.

2. Schnapper A, Becker S., Avsar M., Warnecke G., Ochs M. How to evaluate cellular and subcellular effects of ex vivo lung perfusion quantitatively in a large animal model? Technical procedures to obtain high quality samples for sterological analysis. [www.anatomische-gesellschaft.de](http://www.anatomische-gesellschaft.de) DOI 10.3337/anatges.2012.0017; 2012; p. 69.

3. Mühlfeld C, Knudsen L, Ochs M. Stereology and morphometry of lung tissue. In: Taatjes DJ, Roth J, editors. Cell Imaging Techniques: Methods and Protocols. 2nd ed. New York/Heidelberg: Springer; 2013. p. 367-390.

4. Schneider JP, Ochs M. Alterations of mouse lung tissue dimensions during processing for morphometry: a comparison of methods. Am J Physiol Lung Cell Mol Physiol. 2014;306:L341-L350.

5. Weibel ER, Hsia CC, Ochs M. How much is there really? Why stereology is essential in lung morphometry. J Appl Physiol. 2007;102:459-467.

6. Mühlfeld C, Ochs M. Quantitative microscopy of the lung: a problem-based approach. Part 2: stereological parameters and study designs in various diseases of the respiratory tract. Am J Physiol Lung Cell Mol Physiol. 2013;305:L205-L221.

7. Weibel ER, Knight BW. A morphometric study on the thickness of the pulmonary air-blood barrier. J Cell Biol. 1964;21:367-396.
