# Supplementary material for: Cellular and acellular ex vivo lung perfusion preserve functional lung ultrastructure in a large animal model: a stereological study
Source: Respir Res. 2018 Dec 4;19:238. doi: 10.1186/s12931-018-0942-5 (PMC6278069; doi:10.1186/s12931-018-0942-5)
Supplement: Supplementary file 2 — Table S1. Stereological data on lung structure and alveolar septum composition: absolute volumes and volume densities. Mean ± standard deviation except for 1. Group differences were tested for statistical significance by ANOVA and post hoc Tukey test except for 1. 1 Data deviated from normal distribution and/or homogeneity of variances; thus median ± interquartile range are listed and the Kruskal-Wallis test was used for analysis of group differences. Significance of differences (p < 0.05) is indicated by superscript letters. Groups marked with the same letter do not differ significantly. V absolute volume, VV volume density, npar non-parenchyma, par parenchyma, air alveolar air space, atelect atelectasis, alvsept interalveolar septa, surf surfactant, alvepi alveolar epithelium, septint septal interstitium, capendo capillary endothelium, caplum capillary lumen, bab blood-air-barrier. Table S2. Stereological data on alveolar and capillary surface areas and thickness of blood-air barrier components. Data are given as mean ± SD. Group differences were tested for statistical significance by ANOVA and post hoc Tukey test. Significance of differences (p < 0.05) is indicated by superscripts. Groups marked with the same letter do not differ significantly. S surface area, SV surface density, SS relative surface area, $\overline{\uptau}$τ¯ arithmetic mean thickness, alvepi alveolar epithelium, capendo capillary endothelium, alvepi thin alveolar epithelium of thin bab, bab blood-air barrier, septint septal interstitium, thin bab thin part of bab, thick bab thick part of blood-air barrier. Table S3. Stereological data on oedema parameters. Data are given as median ± interquartile range. Group differences were tested for statistical sign [file 12931_2018_942_MOESM2_ESM.docx]

**Additional file 2**

**Table S1** Stereological data on lung structure and alveolar septum composition: absolute volumes and volume densities

| **Parameter** | **Control** | **Ischaemia** | **aEVLP** | **eEVLP** |
| --- | --- | --- | --- | --- |
| V (lung) [ml] | 1404 ± 478^a^ | 1393 ± 271^a^ | 2233 ± 144^b^ | 1999 ± 294^b^ |
| V (npar, lung) [ml] | 190 ± 34^a^ | 220 ± 26^a^ | 258 ± 26^b^ | 250 ± 64^ab^ |
| V (par, lung) [ml] | 1213 ± 449^a^ | 1173 ± 269^a^ | 1975 ± 132^b^ | 1739 ± 342^ab^ |
| V (air, lung) [ml] | 1061 ± 434^a^ | 986 ± 290^a^ | 1790 ± 151^b^ | 1561 ± 320^ab^ |
| V (atelect, lung) [ml]^1^ | 49.6 ± 91.0^ab^ | 70.7 ± 129.7^a^ | 6.0 ± 54.7^b^ | 27.8 ± 30.2^b^ |
| V (surf, lung) [ml]^1^ | 0.12 ± 0.14 | 0.32 ± 0.91 | 0.69 ± 3.35 | 0.00 ± 0.46 |
| V (alvsept, lung) [ml] | 152 ± 51 | 187 ± 45 | 166 ± 46 | 163 ± 22 |
| V (alvepi, lung) [ml] | 16.75 ± 6.96 | 19.65 ± 11.94 | 35.21 ± 16.14 | 23.28 ± 5.48 |
| V (septint, lung) [ml] | 31.31 ± 15.69 | 50.13 ± 26.07 | 32.76 ± 8.36 | 36.91 ± 3.11 |
| V (capendo, lung) [ml] | 21.18 ± 8.47 | 22.60 ± 6.61 | 24.58 ± 12.19 | 23.29 ± 2.68 |
| V (bab, lung) [ml] | 69.24 ± 29.90 | 92.38 ± 37.04 | 92.54 ± 24.36 | 83.48 ± 4.91 |
| V (caplum, lung) [ml] | 82.44 ± 27.23 | 94.29 ± 33.55 | 73.75 ± 25.33 | 79.92 ± 22.91 |
|  |  |  |  |  |
| V_V_ (npar/lung) | 0.14 ± 0.03 | 0.16 ± 0.03 | 0.12 ± 0.01 | 0.13 ± 0.04 |
| V_V_ (par/lung) | 0.86 ± 0.03 | 0.84 ± 0.03 | 0.88 ± 0.01 | 0.87 ± 0.04 |
| V_V_ (air/lung) | 0.74 ± 0.07 | 0.70 ± 0.07 | 0.80 ± 0.02 | 0.78 ± 0.05 |
| V_V_ (atelect/lung) | 0.024 ± 0.084^ab^ | 0.054 ± 0.107^a^ | 0.002 ± 0.025^b^ | 0.013 ± 0.019^b^ |
| V_V_ (surf/lung) | 0.0001 ± 0.0014 | 0.0003 ± 0.0006 | 0.0003 ± 0.0015 | 0.0000 ± 0.0002 |
| V_V_ (alvsept/lung) | 0.11 ± 0.05 | 0.14 ± 0.04 | 0.08 ± 0.02 | 0.08 ± 0.01 |
| V_V_ (alvepi/lung) | 0.012 ± 0.005 | 0.014 ± 0.009 | 0.016 ± 0.008 | 0.012 ± 0.002 |
| V_V_ (septint/lung) | 0.022 ± 0.008^ab^ | 0.037 ± 0.019^a^ | 0.015 ± 0.003^b^ | 0.019 ± 0.003^ab^ |
| V_V_ (capendo/lung) | 0.015 ± 0.006 | 0.017 ± 0.007 | 0.011 ± 0.006 | 0.012 ± 0.002 |
| V_V_ (bab/lung) [ml] | 0.049 ± 0.018 | 0.068 ± 0.028 | 0.042 ± 0.012 | 0.043 ± 0.006 |
| V_V_ (caplum/lung) | 0.064 ± 0.030 | 0.071 ± 0.035 | 0.034 ± 0.013 | 0.040 ± 0.011 |

Mean ± standard deviation except for ^1^. Group differences were tested for statistical significance by ANOVA and post hoc Tukey test except for ^1^. ^1^ Data deviated from normal distribution and/or homogeneity of variances; thus median ± interquartile range are listed and the Kruskal-Wallis test was used for analysis of group differences. Significance of differences (p<0.05) is indicated by superscript letters. Groups marked with different letters only differ significantly; groups marked with the same letter do not differ significantly. V absolute volume, V_V_ volume density, npar non-parenchyma, par parenchyma, air alveolar air space, atelect atelectasis, alvsept interalveolar septa, surf surfactant, alvepi alveolar epithelium, septint septal interstitium, capendo capillary endothelium, caplum capillary lumen, bab blood-air-barrier.

**Table S2**  Stereological data on alveolar and capillary surface areas and thickness of blood-air barrier components

| **Parameter** | **Control** | **Ischaemia** | **aEVLP** | **eEVLP** |
| --- | --- | --- | --- | --- |
| S (alvepi, lung) [m²] | 61.01 ± 26.01 | 64.71 ± 18.59 | 109.08 ± 54.13 | 73.57 ± 4.48 |
| S (capendo, lung) [m²] | 73.49 ± 28.98 | 74.71 ± 9.12 | 76.14 ± 36.35 | 71.09 ± 15.23 |
|  |  |  |  |  |
| S_V_ (alvepi/lung) | 0.046 ± 0.022 | 0.046 ± 0.018 | 0.049 ± 0.026 | 0.038 ± 0.006 |
| S_V_ (capendo/lung) | 0.055 ± 0.027 | 0.055 ± 0.013 | 0.035 ± 0.017 | 0.036 ± 0.009 |
|  |  |  |  |  |
| S_S_ (alvepi thin/alvepi) | 0.415 ± 0.086 | 0.459 ± 0.057 | 0.309 ± 0.083 | 0.364 ± 0.088 |
|  |  |  |  |  |
| $\overline{}$ (bab) [µm] | 1.044 ± 0.222 | 1.302 ± 0.422 | 1.096 ± 0.334 | 1.174 ± 0.203 |
| $\overline{}$ (alvepi) [µm] | 0.277 ± 0.629 | 0.293 ± 0.144 | 0.339 ± 0.098 | 0.318 ± 0.082 |
| $\overline{}$ (septint) [µm] | 0.478 ± 0.135 | 0.711 ± 0.304 | 0.428 ± 0.228 | 0.520 ± 0.098 |
| $\overline{}$ (capendo) [µm] | 0.289 ± 0.052 | 0.299 ± 0.065 | 0.329 ± 0.064 | 0.336 ± 0.057 |
| $\overline{}$ (thin bab) [µm] | 0.342 ± 0.055 | 0.296 ± 0.089 | 0.333 ± 0.052 | 0.410 ± 0.078 |
| $\overline{}$ (thick bab) [µm] | 1.475 ± 0.342 | 1.968 ± 0.560 | 1.579 ± 0.447 | 1.689 ± 0.170 |

Data are given as mean ± SD. Group differences were tested for statistical significance by ANOVA. No significant (p<0.05) group differences were detected. S surface area, S_V_ surface density, S_S_ relative surface area, $\overline{}$ arithmetic mean thickness, alvepi alveolar epithelium, capendo capillary endothelium, alvepi thin alveolar epithelium of thin bab, bab blood-air barrier, septint septal interstitium, thin bab thin part of bab, thick bab thick part of blood-air barrier.

**Table S3** Stereological data on oedema parameters

| **Parameter** | **Control** | **Ischaemia** | **aEVLP** | **eEVLP** |
| --- | --- | --- | --- | --- |
| V (pbv oed, lung) [ml] | 2.32 ± 6.42^a^ | 2.71 ± 7.82^a^ | 18.59 ± 51.50^b^ | 11.27 ± 58.13^ab^ |
| V (alv oed, lung) [ml] | 0.16 ± 4.07^a^ | 0.36 ± 0.60^a^ | 47.12 ± 49.09^b^ | 9.31 ± 20.38^c^ |
| V_V_ (pbv oed/lung) | 0.0022 ± 0.0044 | 0.0020 ± 0.0069 | 0.0081 ± 0.022 | 0.0046 ± 0.032 |
| V_V_ (alv oed/lung) | 0.0001 ± 0.0038 | 0.0003 ± 0.0003 | 0.0023 ± 0.0215 | 0.0058 ± 0.0094 |
| S (oed-alvepi) [m²] | 0.10 ± 0.69^a^ | 0.65 ± 1.37^a^ | 27.98 ± 8.19^c^ | 4.98 ± 17.71^b^ |
| S_S_ (oed thin/alvepi thin) | 0.01 ± 0.01^a^ | 0.01 ± 0.02^a^ | 0.30 ± 0.31^b^ | 0.10 ± 0.32^ab^ |
| S_S_ (oed thick/alvepi thick) | 0.00 ± 0.01^a^ | 0.00 ± 0.02^a^ | 0.29 ± 0.17^b^ | 0.09 ± 0.24^b^ |
| $\overline{}$ (oedema) [µm] | 0.42 ± 3.01 | 0.53 ± 2.75 | 2.66 ± 5.21 | 2.75 ± 5.37 |

Data are given as median ± interquartile range. Group differences were tested for statistical significance using the Kruskal-Wallis test. Significance of differences (p<0.05) is indicated by superscript letters. Groups marked with different letters only differ significantly; groups marked with the same letter do not differ significantly.

V volume, V_V_ volume density , S surface area, S_S_ relative surface area, $\overline{}$ arithmetic mean thickness, pbv oed peribronchovascular oedema, alv oed alveolar oedema, oed-alvepi alveolar epithelial surface covered with oedema fluid, oed thin alveolar epithelium of the thin blood-air barrier (bab) covered with oedema fluid, alvepi thin alveolar epithelium of thin bab, oed thick alveolar epithelium of the thick bab covered with oedema fluid, alvepi thick alveolar epithelium of thick bab.
